# Supplementary material for: Analysis of PPARγ Signaling Activity in Psoriasis
Source: Int J Mol Sci. 2021 Aug 10;22(16):8603. doi: 10.3390/ijms22168603 (PMC8395241; doi:10.3390/ijms22168603)
Supplement: Supplementary file 1 [file ijms-22-08603-s001.zip › Supplemental materials_Analysis of PPARg signaling activity in psoriasis/Pathway models/Models images and html files/Model of PPARG signaling in psoriasis/9770.html]

PLAAT4


# Protein PLAAT4

|  |  |
| --- | --- |
| URN | urn:agi-llid:5920 |
| Total Entities | 0 |
| Connectivity | 442 |
| Name | PLAAT4 |
| Description | retinoic acid receptor responder (tazarotene induced) 3 |
| Notes | Retinoids exert biologic effects such as potent growth inhibitory and cell differentiation activities and are used in the treatment of hyperproliferative dermatological diseases. These effects are mediated by specific nuclear receptor proteins that are members of the steroid and thyroid hormone receptor superfamily of transcriptional regulators. RARRES1, RARRES2, and RARRES3 are genes whose expression is upregulated by the synthetic retinoid tazarotene. RARRES3 is thought act as a tumor suppressor or growth regulator. [provided by RefSeq, Jul 2008] |

---

|  |  |
| --- | --- |
| Pathway | TLR4 Signaling in Leukocytes |
|  | Dendritic Cell Dysfunction in Crohn's disease |
|  | Model of PPARG signaling in psoriasis |
|  | 1\_Defects in response to pathogens in the gut promotes inflammation in Crohn's disease |

---

|  |  |
| --- | --- |
| MedScan ID | 5920 |

---

|  |  |
| --- | --- |
| LocusLink ID | 5920 |

---

|  |  |
| --- | --- |
| Alias | RIG1 |
|  | TIG3 |
|  | PLAAT4 |
|  | tazarotene-induced gene 3 |
|  | PLA1/2-3 |
|  | RAR-responsive protein TIG3 |
|  | RARRES3 |
|  | MGC8906 |
|  | phospholipase A and acyltransferase 4 |
|  | HRAS-like suppressor 4 |
|  | PLAAT-4 |
|  | retinoid-inducible gene 1 protein |
|  | HRSL4 |
|  | retinoic acid receptor responder protein 3 |
|  | retinoic acid receptor responder (tazarotene induced) 3 |
|  | HRASLS4 |
|  | phospholipase A/acyltransferase-4 |
|  | retinoic acid receptor responder (tazarotene induced) III |
|  | retinoic acid receptor responder 3 |
|  | retinoic acid-inducible gene 1 |
|  | tazarotene-induced gene 3 protein |

---

|  |  |
| --- | --- |
| EC Number | 3.1.1.32 |
|  | 3.1.1.4 |
|  | 2.3.1.- |
|  | 3.1.1.- |

---

|  |  |
| --- | --- |
| GO ID | 0052740 |
|  | 0016410 |
|  | 0052739 |
|  | 0008970 |
|  | 0004623 |
|  | 0102567 |
|  | 0102568 |
|  | 0070292 |
|  | 0016042 |
|  | 0008285 |
|  | 0036152 |
|  | 0006644 |
|  | 0045618 |
|  | 0150074 |
|  | 0005829 |
|  | 0016021 |
|  | 0016020 |

---

|  |  |
| --- | --- |
| KEGG ID | hsa:5920 |

---

|  |  |
| --- | --- |
| Organism | Homo sapiens {Organism urn:agi-taxid:9606} |
|  | Homo sapiens |

---

|  |  |
| --- | --- |
| OMIM ID | 605092 |

---

|  |  |
| --- | --- |
| Hugo ID | 9869 |

---

|  |  |
| --- | --- |
| Human chromosome position | 11q12.3 |
|  | 11q23 |

---

|  |  |
| --- | --- |
| Swiss-Prot Accession | Q9UL19 |
|  | Q9UL19.1 |
|  | B2R599 |
|  | B4DDW2 |
|  | E7ENZ7 |
|  | O95200 |

---

|  |  |
| --- | --- |
| GenBank ID | NC\_000011 |
|  | NM\_004585 |
|  | NP\_004576 |
|  | AP001591 |
|  | CH471076 |
|  | EAW74156 |
|  | KF455398 |
|  | AB030815 |
|  | BAB08109 |
|  | AB453252 |
|  | BAH22446 |
|  | AF060228 |
|  | AAC84000 |
|  | AF092922 |
|  | AAF02294 |
|  | AK293357 |
|  | BAG56873 |
|  | AK312110 |
|  | BAG35046 |
|  | BC009678 |
|  | AAH09678 |
|  | DA973023 |
|  | LC420314 |
|  | BBG06131 |
|  | R55646 |
|  | Q9UL19 |
|  | NC\_018922 |
|  | AMYH02024911 |
|  | DQ891444 |
|  | ABM82370 |
|  | DQ894622 |
|  | ABM85548 |

---

|  |  |
| --- | --- |
| Swiss-Prot ID | PLAT4\_HUMAN |
|  | HRSL4\_HUMAN |

---

|  |  |
| --- | --- |
| Cell Localization | Membrane |

---

|  |  |
| --- | --- |
| Ensembl ID | ENSG00000133321 |
|  | ENSP00000255688.3 |
|  | ENST00000255688.8 |
|  | ENSP00000255688 |
|  | ENST00000255688 |

---

|  |  |
| --- | --- |
| Unigene ID | Hs.17466 |

---
